# Interface Engineering of Organic Schottky Barrier Solar Cells and Its Application in Enhancing Performances of Planar Heterojunction Solar Cells

**DOI:** 10.1038/srep26262

**Published:** 2016-05-17

**Authors:** Fangming Jin, Zisheng Su, Bei Chu, Pengfei Cheng, Junbo Wang, Haifeng Zhao, Yuan Gao, Xingwu Yan, Wenlian Li

**Affiliations:** 1 State Key Laboratory of Luminescence and Applications, Changchun Institute of Optics, Fine Mechanics, and Physics, Chinese Academy of Sciences, Changchun 130033, People’s Republic of China; 2 School of Aerospace Science and Technology, Xidian University, Xi’an 710126, P.R. China; 3 University of Chinese Academy of Sciences, Beijing 100039, People’s Republic of China

## Abstract

In this work, we describe the performance of organic Schottky barrier solar cells with the structure of ITO/molybdenum oxide (MoOx)/boron subphthalocyanine chloride (SubPc)/bathophenanthroline (BPhen)/Al. The SubPc-based Schottky barrier solar cells exhibited a short-circuit current density (Jsc) of 2.59 mA/cm^2^, an open-circuit voltage (Voc) of 1.06 V, and a power conversion efficiency (PCE) of 0.82% under simulated AM1.5 G solar illumination at 100 mW/cm^2^. Device performance was substantially enhanced by simply inserting thin organic hole transport material into the interface of MoOx and SubPc. The optimized devices realized a 180% increase in PCE of 2.30% and a peak Voc as high as 1.45 V was observed. We found that the improvement is due to the exciton and electron blocking effect of the interlayer and its thickness plays a vital role in balancing charge separation and suppressing quenching effect. Moreover, applying such interface engineering into MoOx/SubPc/C_60_ based planar heterojunction cells substantially enhanced the PCE of the device by 44%, from 3.48% to 5.03%. Finally, we also investigated the requirements of the interface material for Schottky barrier modification.

Organic solar cells (OSCs) are a potential inexpensive alternative to conventional inorganic solar cells due to their ease of processing and compatibility with flexible substrates, and have received significant industrial and academic interest as a promising source of inexpensive renewable energy^1^,^2^. Although power conversion efficiency (PCE) of the OSCs is improved continually by designing novel materials and device architectures, is still inadequate in meeting commercial requirements. One of the major challenges preventing OSCs from achieving high efficiency is the low open-circuit voltage (V_OC_) which is restricted by the energy offset between the highest occupied molecular orbital (HOMO) of the electron donor and the lowest unoccupied molecular orbital (LUMO) of the electron acceptor^3^. In 2010, Tang and co-workers reported a kind of organic Schottky barrier photovoltaic cells based on MoOx/C_60_ and they achieved a high Voc of 1.23 V with a simple structure of ITO/MoOx/C_60_/bathophenanthroline (BPhen)/LiF/Al^4^. The PCE of the device is low, only about 0.5% since the weak absorption of the C_60_ and the low FF of the Schottky solar cells. They improved their devices in 2011 by introducing very little donor material (5%, for example) in C_60_ acceptor matrix^5^. This architecture integrated the advantages of both Schottky barrier and donor-acceptor heterojunction photovoltaic cells: Schottky barrier can offer high Voc and donor-acceptor heterojunction can provide more dissociation sites to obtain more photocurrent respectively. Devices based on such architecture achieve PCE beyond 8% were recently reported, showing huge potential of the architecture in achieving high PCEs of the OSCs^6^. With few exceptions, a fullerene-based material such as C_60_ or C_70_ was selected as the acceptor matrix with MoOx to form the Schottky barrier due to their high electron mobility and large exciton diffusion length. Metallophthalocyanines (MPcs) with lower band gap which have been widely used as electron donor material show reasonable ambipolar carrier transporting properties for efficient charge transfer and MPcs have been reported for promise electron acceptor material in OSCs^7^,^8^. It is expectable that Schottky junction solar cells can be designed using MPcs to instead of C_60_, however, to date few devices in such architecture are observed.

In the present study, we examine the performance of Schottky barrier cell by replacing C_60_ with SubPc as the active layer. We obtained a short-circuit current density (Jsc) of 2.59 mA/cm^2^, a Voc of 1.06 V, and a PCE of 0.82%. Device performance was further increased by inserting a thin hole transport layer (HTL) of rubrene into the interface of MoOx/SubPc, and optimized OSCs realize a PCE of 2.30%. The explanation for performance enhancement by including rubrene was demonstrated to be the suppressing of exciton and charge recombination at anode side. When such interface engineering was applied in MoOx/SubPc/C_60_ planar heterojunction OSCs, substantial improvement in the PCE from 3.48% to 5.03% was obtained. The requirements of the interface material as the Schottky barrier modification layer was also invested.

Organic cells with the architecture of ITO/MoOx (5 nm)/rubrene/SubPc (45 nm)/BPhen (6 nm)/Al (80 nm) were fabricated with various thicknesses of rubrene layer. Device structure and the energy level diagram are shown schematically in Fig. 1. Figure 2 depicts the current density versus voltage (J–V) characteristics of the devices with various thicknesses of rubrene of 0, 2, 5, 10 and 20 nm. Devices numerical values derived from J–V curves are summarized in Table 1. The organic Schottky solar cells based on SubPc output a Jsc of 2.59 mA/cm^2^ which is the double magnitude of those Schottky solar cells with fullerene as the active layer and should be ascribed to the very strong absorption coefficient of SubPc^4^. The relative large Jsc also shows that the Fermi energy level difference between ITO/MoOx anode and SubPc is high enough to separate excitons generated in SubPc. The device behaves typical Schottky-type photovoltaic characteristics with a low FF. Although the thickness of SubPc is 45 nm which is threefold of that when it is employed as donor in a planar heterojunction cells, exciton separation only occurs near Schottky junction, which is determined by the building-in electric field and the exciton diffusion length of SubPc. For instance, only excitons that generated within about 10 nm apart from the junction can be separated for the C_60_-based Schottky cell^9^.

The photovoltaic response of SubPc based Schottky barrier cell is substantially improved by inserting rubrene into the cell. For the best cell with 5 nm rubrene, PCE increased by 180%, from 0.82% to 2.30% with Jsc from 2.59 to 4.17 mA/cm^2^, Voc from 1.06 to 1.35 V and FF from 0.30 to 0.41. It can be seen that photocurrent strongly depends on the thickness of rubrene and reaches its maximum at 5 nm rubrene, then decreases with further increasing the thickness. All five cells display high Voc which is increased continuously with raising rubrene thickness. The highest Voc obtained is 1.45 V which, to the best of our knowledge, is the highest one reported of the OSCs without a tandem design.

Several possible reasons for the improvement of the photoelectric response by inserting thin rubrene are proposed to be considered.

We firstly think about the possibility of device work mechanism changes from Schottky barrier cell to donor/acceptor planar heterojunction and inserted rubrene provides a donor/acceptor interface of rubrene/SubPc. It is in doubt that whether there is a fundamental correlation between the exciton binding energy and the energy offset needed to ensure efficient charge transfer (CT) at the donor/acceptor interface, the opinion of that to obtain a required photoinduced charge transfer, LUMO (or HOMO) offset between donor and acceptor must be larger than the exciton binding energy in the donor (or acceptor) is still commonly accepted. For the pair of rubrene/SubPc, the HOMO offset is only about 0.2 eV and the reported exciton binding energy of SubPc is 0.4 eV^10^, which could prevent the dissociation of the exciton generated in SubPc. There have been, however, a few reports indicating that SubPc can be used as acceptor composed with some donors with small energy offset. In 2012, for example, Nicola Beaumont etc. reported a planar heterojunction OSC with tetracene (Tc) as the donor and SubPc as the acceptor, obtaining a relative high FF of 0.58 and a PCE of 2.9%^11^. In our case here, is there a possibility that rubrene and SubPc constitute an effective heterojunction for exciton dissociation?

IPCE characteristics of the devices are presented in Fig. 3a. The maximum photoelectric response is obtained at a 5 nm rubrene, with an IPCE = 45% at 590 nm corresponding to the absorption peak of SubPc. In accordance to the decreased Jsc, photoelectric response declines with increasing rubrene thickness, which means that thicker rubrene is less than effective and internal quantum efficiency (IQE) and/or optical absorption efficiency reach their/its maximum at a 5 nm rubrene layer. More importantly, compare the IPCE characteristics to the absorption spectra presented in Fig. S1, a significant fraction of the photocurrent is contributed by the SubPc layer and contribution from rubrene can hardly be distinguished. This implies that rubrene/SubPc is not an effective dissociation heterojunction at least for exciton generated in rubrene in despite of that the LUMO offset between rubrene and SubPc is 0.4 eV which is the double of their HOMO offset.

Figure 3b shows the Jsc and FF as functions of rubrene thickness at 45 nm SubPc. Both Jsc and FF have a strong dependence on rubrene thickness, and the optimal thickness for rubrene is only 5 nm which is in big difference with 60 nm Tc as donor with SubPc as acceptor of the devices in the reference^11^. As we know, in OSCs, the optimal thickness of organic active layers is strongly dependent on their mobilities and exciton diffusion lengths (L_D_). The L_D_ of rubrene and optimal thickness in planar heterojunction OSCs with C_60_ as acceptor were reported to be about 10 nm and 20 nm, respectively^12^,^13^. Interestingly, the optimized thickness of rubrene is as thin as 5 nm for present SubPc-based OSC devices and a thickness of 20 nm rubrene seriously damages the total performances. Confusion exists that why optimum thickness for rubrene is only 5 nm if rubrene and SubPc construct effective dissociation junction ?

We also demonstrated the devices with DBP as the interlayer to replace rubrene (Fig. S2). Selecting DBP comes from two considerations. One is that HOMO of DBP locates more closely with SubPc and there is a smaller offset (less than 0.1 eV) for excitons generated in SubPc to separate at such interface. The other reason is that DBP has a strong absorption beyond 600 nm where the absorbance of SubPc is rather weak and photocurrent contribution from DBP can be easily recognized from the IPCE spectra. However, almost the same regularity was observed by replacing rubrene with DBP as the interlayer that optimum 5 nm DBP gave a great improvement for cell performance and photocurrent contribution from absorption of DBP is not obtained. From the above discussions, the mechanism of that the added donor-acceptor interface enhances device performance is likely inappropriate.

Secondly, we consider the possibility of that absorption of SubPc is enhanced by introducing rubrene, which may result from the structural template effect of rubrene on SubPc. SubPc has a non-planar molecule structure and can pack in different orientations by change the deposition conditions. Actually, reports of the structural templating effect increasing the absorption for metallophthalocyanines have been widely reported^14^,^15^. The growing of molecules changes from standing up orientation to horizontal orientation by introducing the structural templating interlayer such as CuI or 3,4,9,10-perylene tetracarboxylic acid (PTCDA) giving rise to an increase in absorption coefficient^14^,^16^. We tested the absorption spectra of the device with or without rubrene interlayer to verify the hypothesis. The absorption spectra are given in Fig. 4. As show in Fig. 4, the absorption spectrum changes little and great absorption enhancement of SubPc is not observed, which implies that the hypothesis that rubrene has a structural template effect on SubPc is not correct.

In a typical structure of the OSCs, exciton blocking layers (EBL) can play a vital role for achieving efficient OSCs as its important function in protecting excitons and charges from reaching to electrode surface, thus avoiding quenching of the excitons and charges by the electrode surface states. Cathode EBLs have been widely studied and different EBLs are successfully applied in OSCs^17^,^18^,^19^,^20^,^21^. Researchers also found that the anode or the anode buffer layer such as Poly(3,4-ethylene dioxythiophene): poly(styrene sulfonate) (PEDOT:PSS) quenches excitons^22^. However, compared to the widely study of the cathode EBLs, there are only few reports about anode EBLs^23^,^24^. In the case here, we doubt that if the ITO/MoOx anode has a quench effect on the exciton generated in SubPc in spite of that they offer the driving force for exciton dissociation. In order to evaluate quench effect of ITO/MoOx, photoluminescence measurement was applied and the photoluminescence results are provided in Fig. 5. As shown in Fig. 5, SubPc displays a PL emission peak at about 625 nm, which is in accordance with result reported in reference^25^. With inserting 2 nm rubrene between MoOx and SubPc, the PL emission of SubPc shows an obvious increase compared with the single SubPc layer without rubrene, which demonstrates that the inclusion of rubrene does nothing for improving exciton separation and on the contrary, it works as an exciton blocking layer by suppressing the quenching (or dissociation) of excitons at ITO/MoOx. It is no surprise that widely employed ITO anode or anode buffer of MoOx is an effective exciton quencher. In the reference^23^, authors found that even the quartz substrate acts as an exciton quencher. With further increasing the thickness of rubrene, PL emission increases gradually indicating that rubrene does suppress the quenching (or dissociation) of excitons at ITO/MoOx and the suppressing effect is strengthened with increasing the thickness of rubrene. Such blocking effect is double-edged sword: for the one hand it reduces exciton loss and on the other hand it blocks exciton to diffuse to the Schottky junction and decreases the separation efficiency. The blocking effect of rubrene can perfect explain device performance transformation by introducing rubrene. Suppressing recombination results in a lower Js, and hence a high Voc, as Voc of OSCs is approximated determined by Voc = nk_B_T/q ln (Jsc/Js), where n is diode ideality factor, k_B_ is Boltzmann’s constant, T is temperature, q is the fundamental charge, Jsc is the short circuit current density, and Js is the saturation dark current density. On the other hand the less exciton quenching, the more photocurrent collected and an increased Jsc and FF. For a thicker rubrene, it decreases the building-in electric field in SubPc and declines the separation efficiency of exciton, resulting in a decreased Jsc and FF. As a result, there exists a tradeoff between suppressing exciton quenching and promoting exciton separating, and they give a good balance at the best rubrene thickness of 5 nm.

It is necessary to point out that device performance improvement also benefit from the electron blocking effect of the interlayer by decreasing the possibility of the recombination for the electron and hole at the anode side. Since the impressive electron transport characteristic of SubPc, electron-hole recombines severely at anode side and inserting rubrene can block the diffusion of electron to anode side due to its relative shallower LUMO energy and lower electron mobility compared with those of SubPc. As show in the device dark current of Fig. 5b, the dark current of device is significantly suppressed and the rectification ration increase from the order of 10–10^3^ at ±1.5 V by inserting the blocking layer of 5 nm rubrene. The vibration of device dark current is consistent with the change of Voc which is increased with the thickness of rubrene. The inclusion of rubrene suppresses the recombination and leads to a lower Js, and hence a high Voc. Such suppresses effect is increased with the thickness of rubrene and results in the large variation of Voc.

The above results simulate us to reconsider the role of MoOx/SubPc Schottky barrier which is universally used in OSCs. We deduce that exciton quench and charge recombination also exist in planar OSCs and device performance can be improved by interface engineering in Schottky barrier cell. Experimental results demonstrate our speculation. We fabricated the organic planar heterojuction cell with the structure of ITO/MoOx (5 nm)/rubrene/SubPc (15 nm)/C_60_ (40 nm)/ BPhen (6 nm)/Al (100 nm). As show in Fig. 6 and the photovoltaic parameters summarized in Table 2, the reference device without rubrene anode buffer layer of the planar heterojunction OSC offers a Jsc = 5.32 mA/cm^2^, Voc = 1.05 V, FF = 0.62, and PCE = 3.48%, which is comparable with other reports^25^,^26^. Inserting rubrene can significantly improve Jsc, compared with the reference device without rubrene anode EBL, reaching to >7 mA/cm^2^. The best performance is achieved at an optimum rubrene thickness of 2 nm with the performance of J_SC_ = 7.33 mA/cm^2^, V_OC_ = 1.09 V, FF = 0.54 and PCE = 4.37% which offers a 25% increase than that of the reference cell. Unlike Schottky barrier cell, a thicker rubrene can also deliver a high photocurrent while for the Schottky barrier cell thick rubrene is detrimental to the photocurrent. This change lies in the fact that in Schottky barrier cells, thick rubrene decreases the build-in electric field between MoOx and SubPc which decreases exciton dissociation efficiency, while for planar heterojunction cells there have an added SubPc/C_60_ interface and excitons within the diffusion length in SubPc can diffuse to such interface to form photocurrent.

To study the requirements to the inserting hole transport layer (HTL), we tested six donor materials, including rubrene, zinc phthalocynine(ZnPc), 2-n aphthyl(phenyl)amino]tri-phenylamine (2-TNATA), 4,4′,4′′-Tris(N-3-methylphenyl-N-phenyl-amino) triphenylamine (2-MTDATA), 1,1-Bis-4-bis4-methyl-phenyl-amino-phenyl-cyclohexane (TAPC) and 4,4′-Bis(carbazol-9-yl)-biphenyl (CBP). Thin HTLs of 2 nm were used for all the devices for the consideration of favoring hole collecting. The J-V curves and photovoltaic parameters are offered in Fig. 7 and Table S2. As shown in Fig. 7, the devices can be divided to two distinct groups according to their photovoltaic performance. The one group is with rubrene, TAPC and CBP as the interlay and the other one is with ZnPc, 2-TNATA and m-MTDATA. In the former group, device Jsc and PCE increases to >7 mA/cm^2^ and >4.2% respectively by including the HTLs and the best one is CBP-based device with a PCE of 5.03%. In the latter group, device Jsc and PCE decrease to about 4 mA/cm^2^ and <2% by including the HTLs and the worst one is ZnPc-based device with a PCE of less than 1%. It is found that device performances strongly depend on the HOMO value of the HTL. As shown in Fig. S5, HTLs in the former group all have a lower HOMO value, while HTLs in the latter group all have a higher HOMO value. The HOMO dependence can be easily understood by taking the energy match of the HTLs with MoOx into account. For the HTLs in the latter group, their HOMOs are far away from the conduction band of MoOx which precludes the hole transport via the deep-lying gap state that close to the conduction band of MoOx and degrades device Jsc and FF severely^28^,^29^. The Voc of devices with the HTLs of ZnPc, 2-TNATA and m-MTDATA is also much smaller than that of the devices with HTLs of rubrene, TAPC and CBP. For the devices with the interlayers of ZnPc, 2-TNATA and m-MTDATA, the lower HOMO induced an energy barrier for hole collection by ITO/MoOx, which results in accumulation of hole and an increased recombination of electron and hole at anode side. The increased recombination is in agreement with the lower FF and leads to a high Js and hence a low Voc, as Voc of OSCs is approximated determined by Voc = nkBT/q ln (Jsc/Js) as discussed above.

In summary, we describe the performance of organic Schottky barrier cell based on SubPc as the active layer. Device performances were increased 180% by introducing thin hole transport layer at the junction between MoOx and SubPc, which is proved to ascribed to the exciton and electron blocking effect. A maximum Voc as high as 1.45 V is obtained with a single-cell Schottky barrier solar cell. Applying such method into the planar heterojunction cell results in a PCE increase by 44% from 3.48% to 5.03% with a thin CBP as the interlayer.

## Experimental Section

**Figure 1:**
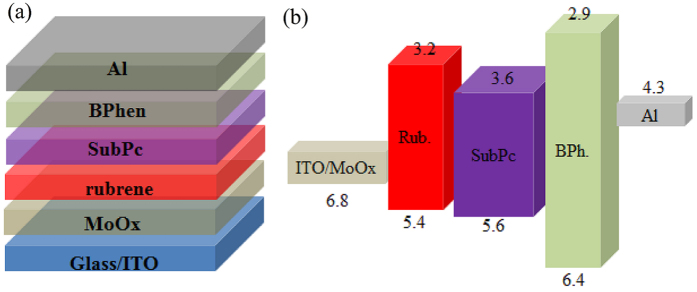
(**a**) Device architecture of the solar cells, (**b**) Schematic energy level diagram.

All organic materials were obtained from a commercial source and were used without further sublimation. Organic solar cells with the architecture of ITO/MoOx (5 nm)/rubrene/SubPc (45 nm)/BPhen (6 nm)/Al (80 nm) were fabricated with various thicknesses of rubrene layer. Device structure and the energy level diagram are shown schematically in Fig. 1. SubPc was used for its considerable absorption and reasonable electron carrier transporting property. Thermally evaporated deposition MoOx is used due to its high workfunction and BPhen is used as cathode buffer layer as usual. For cell fabrication, indium tin oxide (ITO)-coated glass substrates were used (Rs = 15 Ω/sq) after cleaning by ultra-sonication in acetone, detergent, and acetone and exposed to ultraviolet zone for 10 min. All the layers of the OSC devices were successively deposited on ITO glass substrate by thermal evaporation under high vacuum conditions (around 10^−4^ Pa) without breaking the vacuum. The evaporation rates were kept at 1 Å/s for the MoOx and organic layers, and 5 Å/s for the Al cathode. Active areas of the cells were 0.1 cm^2^ defined by the overlapping area of the ITO and Al electrodes.

Current–voltage traces were obtained with a Keithley 2400 source meter in dark and illuminated with a Xe lamp with an AM 1.5 G filter, and the irradiation intensity was certified to be 100 mW/cm^2^. The incident photon to current conversion efficiency (IPCE) spectra were performed with a Stanford SR803 lock-in amplifier under monochromatic illumination at a chopping frequency of 110 Hz by a Stanford SR540 chopper. Absorption spectra were measured with a Shimadzu UV-3101PC spectrophotometer. Steady-state photoluminescent (PL) spectra were measured with a Hitachi F7000 fluorescence spectrophotometer. All measurements were performed under ambient conditions at room temperature with unpackaged devices.

## Additional Information

**How to cite this article**: Jin, F. *et al*. Interface Engineering of Organic Schottky Barrier Solar Cells and Its Application in Enhancing Performances of Planar Heterojunction Solar Cells. *Sci. Rep.* **6**, 26262; doi: 10.1038/srep26262 (2016).

## Supplementary Material

Supplementary Information

## Figures and Tables

**Figure 2:**
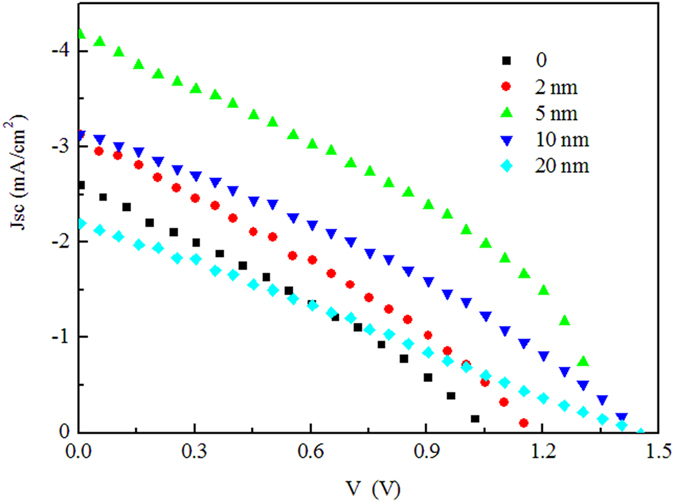
J–V characteristics of solar cells of ITO/MoOx (5 nm)/rubrene/SubPc (45 nm)/BPhen (6 nm)/Al (80 nm) with different thickness of rubrene under 1 sun, AM 1.5G illumination.

**Figure 3:**
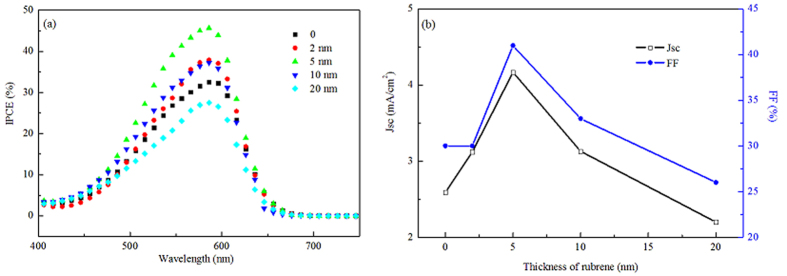
(**a**) IPCE characteristics of the devices with various rubrene thickness. (**b**) Jsc and FF as functions of rubrene thickness.

**Figure 4:**
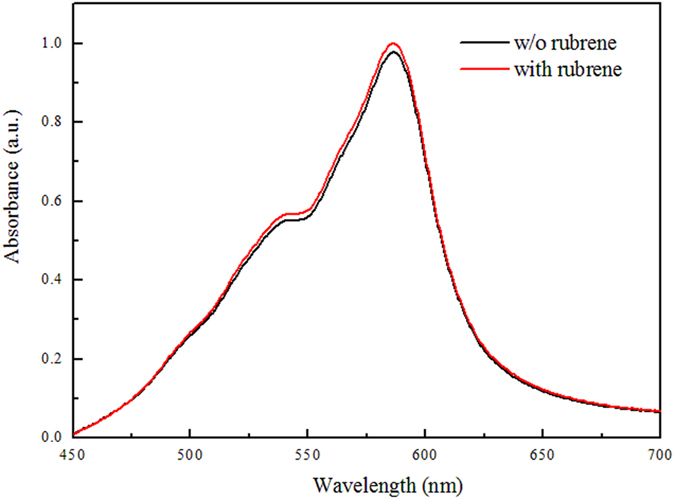
Comparison of absorption spectra of the devices ITO/MoOx (5 nm)/rubrene/SubPc (45 m)/BPhen (6 nm) with or without 5 nm rubrene interlayer.

**Figure 5:**
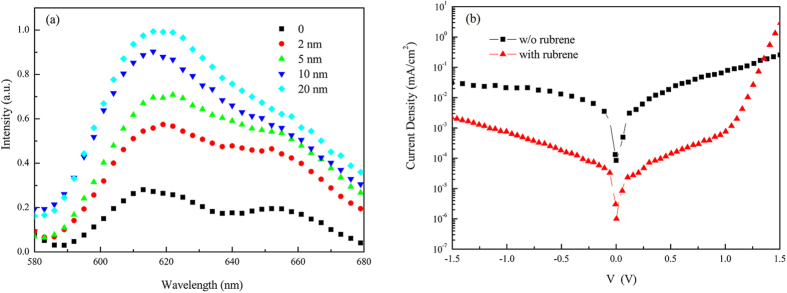
(**a**) Steady-state photoluminescent (PL) spectra of SubPc with different thickness of rubrene on ITO/MoOx substrates for a pump wavelength of 550 nm at room temperature. (**b**) Current density vs voltage characteristics in dark of an ITO/MoOx (5 nm)/rubrene (0 or 5 nm)/SubPc (45 nm)/BPhen (6 nm)/Al (80 nm) cell with or without rubrene blocking layer.

**Figure 6:**
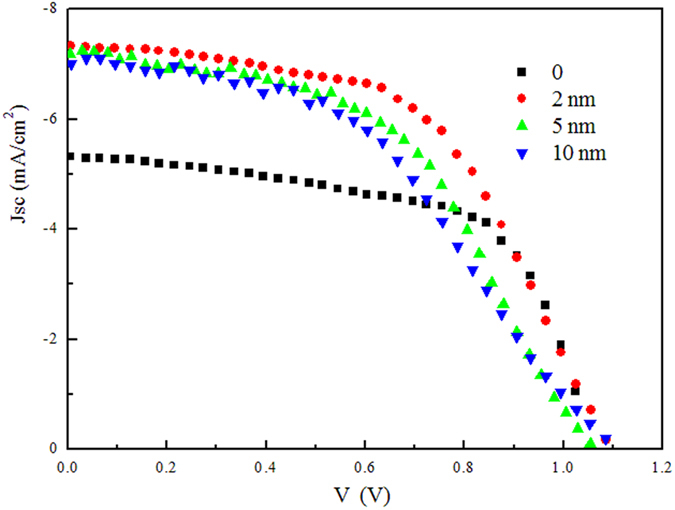
J–V curves of the OSCs of ITO/MoOx (5 nm)/rubrene/SubPc (15 nm)/C_60_ (40 nm)/BPhen (6 nm)/Al (100 nm) with various rubrene thickness under AM 1.5 G solar illumination at 100 mW/cm^2^.

**Figure 7:**
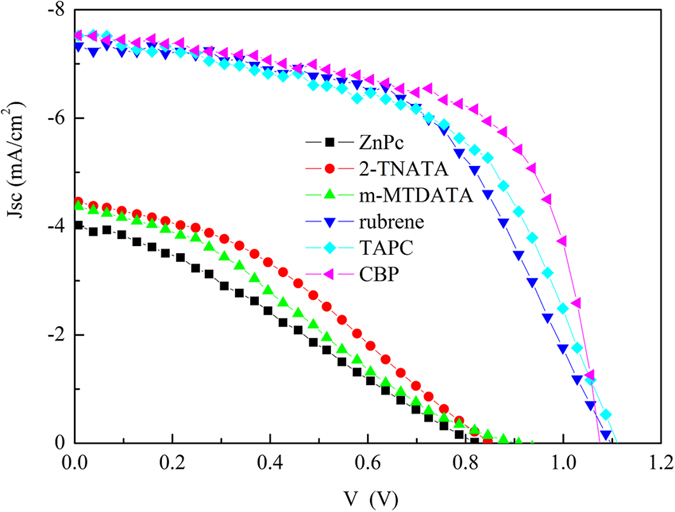
J–V curves of the OSCs with different inserting HTLs in a device structure of ITO/MoOx (5 nm)/HTL (2 nm)/SubPc (15 nm)/C_60_ (40 nm)/ BPhen (6 nm)/Al (100 nm).

**Table 1:** Photovoltaic parameters of the OSC devices with various rubrene thicknesses.

**Thickness of rubrene (nm)**	**J**_**SC**_ **(mA/cm**^2^)	**Voc (V)**	**FF**	**PCE (%)**
0	2.59 ± 0.12	1.06 ± 0.02	0.30 ± 0.02	0.82 ± 0.11
2	3.12 ± 0.14	1.17 ± 0.02	0.30 ± 0.02	1.09 ± 0.12
5	4.17 ± 0.15	1.35 ± 0.03	0.41 ± 0.02	2.30 ± 0.19
10	3.13 ± 0.17	1.43 ± 0.04	0.33 ± 0.03	1.47 ± 0.25
20	2.20 ± 0.19	1.45 ± 0.03	0.26 ± 0.03	0.83 ± 0.19

**Table 2:** Summary of the planar heterojunction cell performances with different thickness of rubrene layer.

**Thickness of rubrene(nm)**	**Jsc (mA/cm**^2^)	**Voc (V)**	**FF**	**PCE (%)**
0	5.32 ± 0.12	1.05 ± 0.02	0.62 ± 0.01	3.48 ± 0.18
2	7.33 ± 0.14	1.09 ± 0.02	0.54 ± 0.01	4.37 ± 0.22
5	7.18 ± 0.15	1.07 ± 0.03	0.50 ± 0.01	3.81 ± 0.27
10	7.01 ± 0.20	1.11 ± 0.04	0.45 ± 0.02	3.54 ± 0.31
