# Supplementary material for: Interface Engineering of Organic Schottky Barrier Solar Cells and Its Application in Enhancing Performances of Planar Heterojunction Solar Cells
Source: Sci Rep. 2016 May 17;6:26262. doi: 10.1038/srep26262 (PMC4869098; doi:10.1038/srep26262)
Supplement: Supplementary Information [file srep26262-s1.doc]

**Interface Engineering of Organic Schottky Barrier Solar Cells and Its Application in Enhancing Performances of Planar Heterojunction Solar Cells**

Fangming Jin^1^, Zisheng Su[[1]](#footnote-2), Bei Chu^1^, Pengfei Cheng^2^, Junbo Wang^1^, Haifeng Zhao^1^, Yuan Gao^1,3^, Xingwu Yan^1,3^**&** Wenlian Li^1^


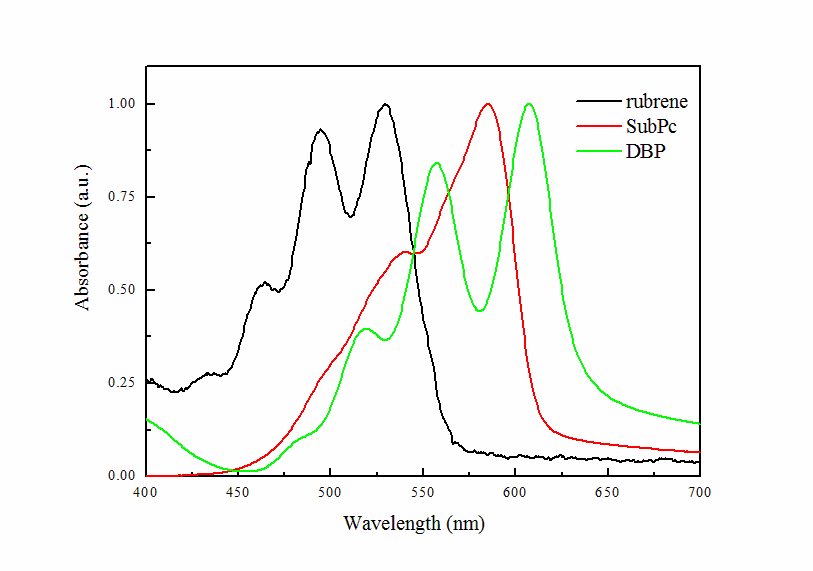


**Figure S1**. Normalized absorption spectra for rubrene, SubPc and DBP.


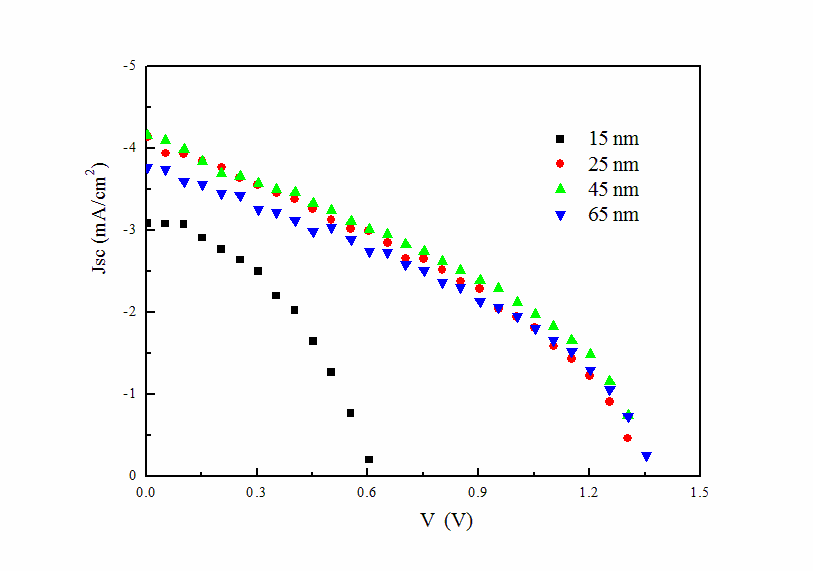


**Figure S2**. J-V characteristics of solar cells of ITO/MoOx (5 nm)/ rubrene (5 nm)/ SubPc/BPhen (6 nm)/Al (80 nm) with different thickness of SubPc under 1 sun, AM 1.5G illumination. Device performances show relatively insensitive to the thickness of the SubPc with the exception of the thinnest 15 nm SubPc device due to its low Voc. FFs change little with increasing the thickness SubPc, which illustrates that the low electron mobility of SubPc does not impact device performance seriously.

**Table S1**. Performances for devices of ITO/MoOx (5 nm)/ rubrene (5 nm)/ SubPc/BPhen (6 nm)/Al (80 nm) with different thickness of SubPc under 1 sun, AM 1.5G illumination.

| **Thickness of SubPc (nm)** | **Jsc (mA/cm^2^)** | **Voc (V)** | **FF** | **PCE (%)** |
| --- | --- | --- | --- | --- |
| **15** | 3.08 | 0.62 | 0.42 | 0.80 |
| **25** | 4.13 | 1.33 | 0.40 | 2.19 |
| **45** | 4.15 | 1.35 | 0.41 | 2.30 |
| **65** | 4.14 | 1.37 | 0.39 | 2.21 |


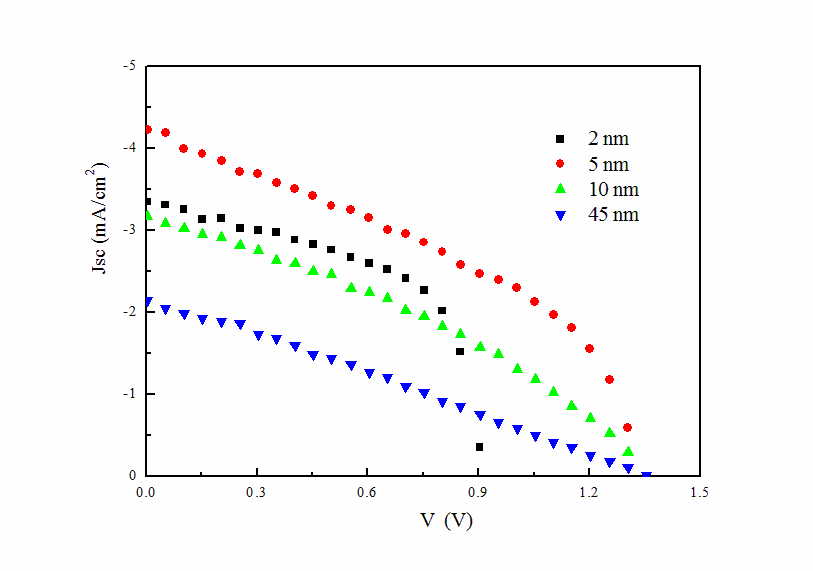


**Figure S3**. J-V characteristics of solar cells of ITO/MoOx (5 nm)/ DBP/ SubPc (25 nm)/BPhen (6 nm)/Al with varying the thickness of DBP. The best device was obtained at a DBP thickness of 5 nm as same as the device of the rubrene-based device.


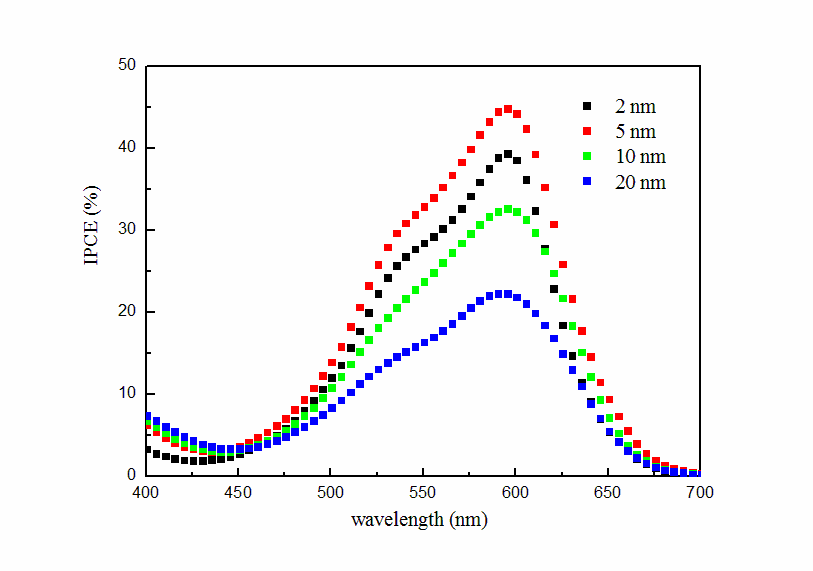


**Figure S4**. IPCE characteristics of the devices with a structure of ITO/MoOx (5 nm)/ DBP/SubPc (25 nm)/BPhen (6 nm)/Al (80 nm) with different thickness of DBP. The absorption peak of DBP at about 610 nm can hardly be distinguished from the IPCE spectra.


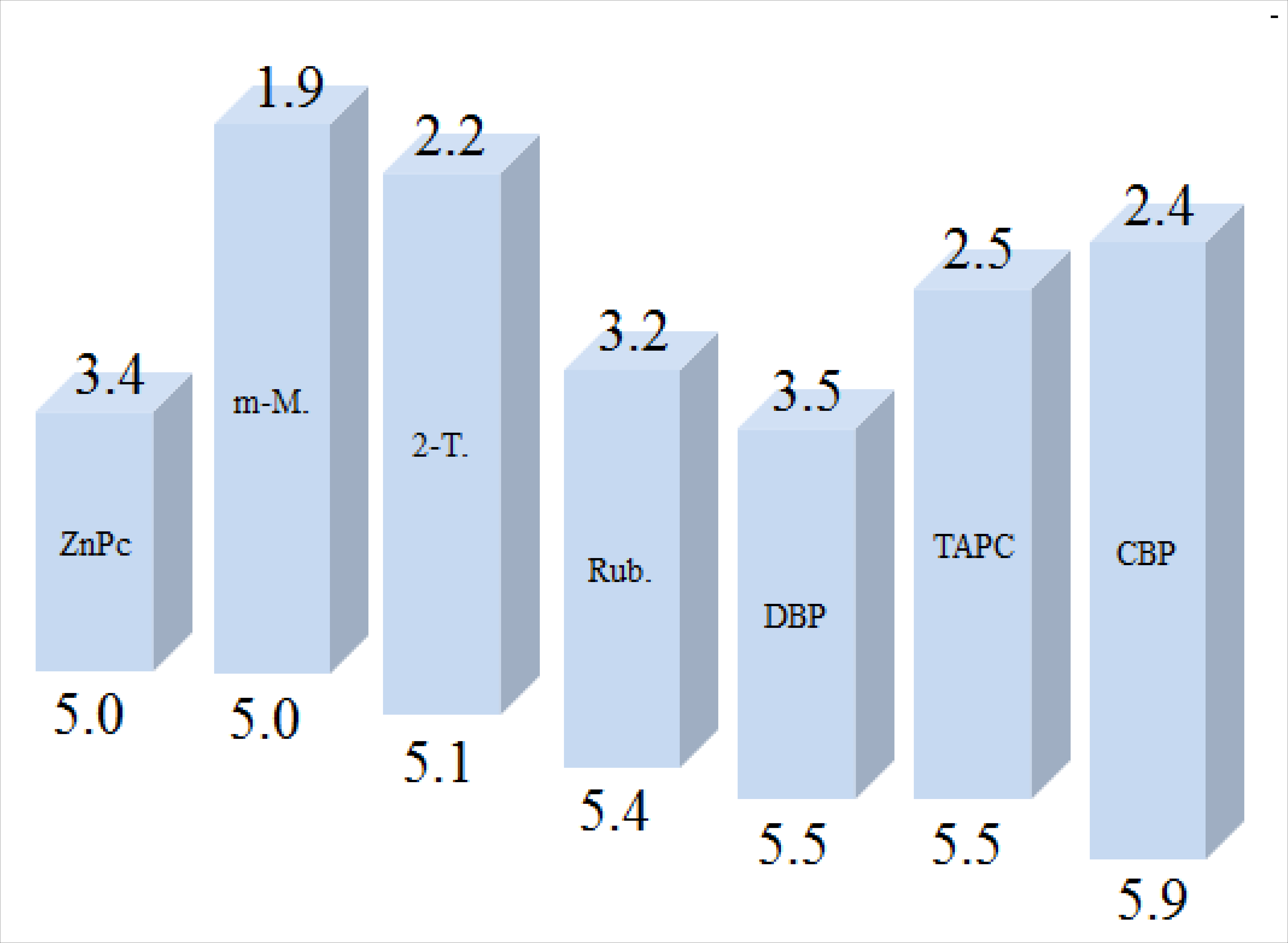


**Figure S5.** Energy level diagram of the HTLs of ZnPc, m-MTDATA, 2-TNATA, rubrene, DBP, TAPC and CBP.

**Table S2**. Performances for devices of ITO/MoOx (5 nm)/ HTL (2 nm)/ SubPc (15 nm)/BPhen (6 nm)/Al (80 nm) with different HTLs under 1 sun, AM 1.5G illumination.

| **HTL (2 nm)** | **Jsc**  **(mA/cm^2^)** | **FF** | **Voc**  **(V)** | **PCE**  **(%)** |
| --- | --- | --- | --- | --- |
| **ZnPc** | 4.02 | 0.29 | 0.82 | 0.96 |
| **2-TNATA** | 4.46 | 0.35 | 0.85 | 1.35 |
| **m-MTDATA** | 4.37 | 0.28 | 0.90 | 1.10 |
| **rubrene** | 7.33 | 0.54 | 1.09 | 4.37 |
| **TAPC** | 7.52 | 0.53 | 1.10 | 4.43 |
| **CBP** | 7.53 | 0.62 | 1.07 | 5.03 |


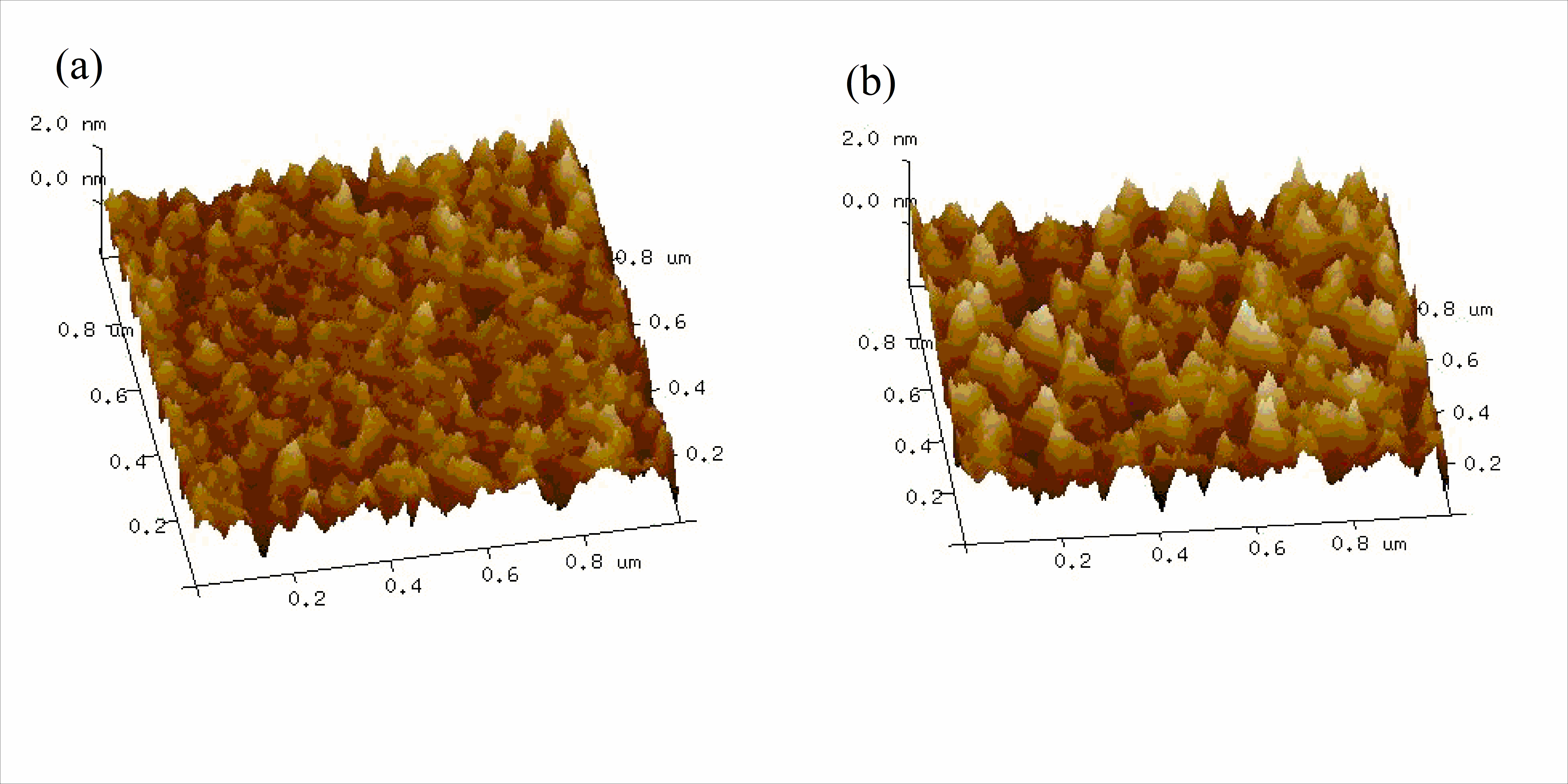


**Figure S6**. AFM images of SubPc (15 nm) with 0 (a) and 2 nm (b) CBP interlayer on Si wafer. AFM images show that both films of SubPc is rather smooth with a root-mean-square roughness (RMS) below 0.5 nm and prominent changes in the surface morphology of SubPc is not occurred.

1. State Key Laboratory of Luminescence and Applications, Changchun Institute of Optics, Fine Mechanics, and Physics, Chinese Academy of Sciences, Changchun 130033, People’s Republic of China. ^2^School of Aerospace Science and Technology, Xidian University, Xi'an 710126, P. R China. ^3^University of Chinese Academy of Sciences, Beijing 100039, People’s Republic of China. Correspondence and requests for materials should be addressed to Z.S. (email:suzs@ciomp.ac.cn) [↑](#footnote-ref-2)
